# Insufficient or Excessive Niacin Intake Induced Glucose and Lipid Metabolic Disorders and Impaired Liver Health in Nile Tilapia

**DOI:** 10.1155/anu/3445390

**Published:** 2026-01-04

**Authors:** Ruixin Li, Jingwen Liu, Jiayong Liang, Lang Liang, Zexuan Kong, Tan Liu, Xiaojuan Liu, Fan Lin, Cuiying Chen, Zhenyu Du, Shuqi Wang

**Affiliations:** ^1^ Guangdong Provincial Key Laboratories of Marine Biotechnology, Shantou University, Shantou, 515063, China, stu.edu.cn; ^2^ LANEH, School of Life Sciences, East China Normal University, Shanghai, 200241, China, ecnu.edu.cn

**Keywords:** hypoglycemia, lipid accumulation, liver health, niacin, Nile tilapia

## Abstract

Niacin (vitamin B3) is involved in the metabolic regulation of energy metabolism in animals. However, both deficiency and excess supplementation of niacin can induce profound physiological disturbances in fish. The present study investigated the effect of niacin on energy metabolism and liver health in Nile tilapia (*Oreochromis niloticus*). Thus, Nile tilapias were fed diets with different niacin supplementation levels (0, 50, 100, and 200 mg/kg) for 8 weeks. The results showed that compared with the niacin‐free group, dietary niacin supplementation (50–200 mg/kg) significantly promoted the growth of tilapia. However, excessive niacin supplementation (200 mg/kg) resulted in significantly higher condition factor and viscerosomatic index (VSI) compared to the moderate supplementation groups (50–100 mg/kg). Accordingly, hepatic triglyceride (TG) content was significantly elevated in the excessive niacin group (200 mg/kg). Furthermore, excessive niacin caused hepatic lipid accumulation by enhancing lipogenesis and inhibiting lipid breakdown, as evidenced by the significantly increased the expression of lipogenic genes while suppressing lipolysis and autophagy. Additionally, serum glucose and liver pyruvate in the niacin‐free group was significantly lower than in other groups. The key genes of glycolysis and gluconeogenesis were significantly downregulated in the liver of Nile tilapia fed with a niacin‐free diet. In contrast, excessive niacin supplementation (200 mg/kg) significantly suppressed hepatic tricarboxylic acid (TCA) cycle‐related gene expression, indicating the inhibition of glucose oxidation for energy production. Further analysis of differential metabolites showed that excessive niacin caused accumulation of oxaloacetate, aspartate, and glutathione, but reduced glutamate content. Additionally, tilapia fed with moderate niacin supplementation (50–100 mg/kg) exhibited significantly lower serum alanine aminotransferase enzyme activity compared to both the niacin‐free and excessive niacin groups. Hepatic catalase (CAT) and superoxide dismutase (SOD) activities were significantly elevated in this group relative to other groups. Overall, the data suggested that niacin deficiency induced hypoglycemia and reduced glucose activity in Nile tilapia, while excessive niacin led to hepatic lipid accumulation. Both deficient and excessive niacin intake compromised liver health and diminished antioxidant capacity (AOC) in Nile tilapia.

## 1. Introduction

Niacin (vitamin B3), serving as the essential biosynthetic precursor for nicotinamide adenine dinucleotide (NAD+) and its phosphorylated form (NADP+) [1], occupies a central position in cellular bioenergetics through its fundamental role in the tricarboxylic acid (TCA) cycle and direct involvement in the metabolic regulation of lipids, proteins, and carbohydrates in aquatic species [2]. Comparative nutritional studies have revealed remarkable interspecies variability in niacin requirements, ranging from 10 mg/kg diet for rainbow trout (*Oncorhynchus mykiss*) to 121 mg/kg for Mozambique tilapia (*Tilapia mossambica*) [3, 4]. Optimal niacin supplementation has been demonstrated to significantly enhance growth and feed conversion ratios across various fish and crustacean species [5]. However, both deficiency and excess supplementation of niacin can induce profound physiological disturbances in aquatic organisms.

The deficiency syndrome manifests through conserved symptoms including anorexia, growth retardation, and elevated mortality, along with species‐specific pathological manifestations [6]. For example, juvenile grass carp (*Ctenopharyngodon idella*) developed hematopoietic suppression and immune compromised states [7]; common carp (*Cyprinus carpio*) exhibited dermal and fin erosion with hemorrhagic presentations [8]; while juvenile largemouth bass (*Micropterus salmoides*) demonstrated compromised intestinal barrier function and defective immune responses [9]. Conversely, excessive niacin intake induced distinct metabolic perturbations, as evidenced in gilthead seabream (*Sparus aurata*) showing dose‐dependent increases in whole‐body lipid deposition culminating in growth suppression and hepatic steatosis under excessive supplementation conditions [10]. Despite these empirical observations, current research exhibits significant mechanistic limitations. The current studies mainly focus on phenotypes (growth performance and immune capacity), but lack in‐depth research on the molecular mechanisms of niacin on metabolism regulation. This knowledge gap substantially impedes the scientific optimization of niacin supplementation strategies in aquafeeds, potentially leading to nutritional imbalances in cultured species [11].

Recent advances in mammalian systems have elucidated multiple regulatory mechanisms of niacin in energy metabolism. In lipid homeostasis, niacin exerted dual modulation by suppressing hepatic lipogenesis while enhancing β‐oxidation in rat hepatocytes, coupled with inhibition of diacylglycerol acyltransferase (DGAT)‐mediated lipid droplet formation [12–14]. Regarding glucose metabolism, niacin improved glycemic control through PPARγ‐mediated transcriptional regulation and GPR109‐dependent signaling in pancreatic β‐cells [15]. Intriguingly, emerging evidence suggested functional conservation of these pathways in aquatic species: Niacin regulates cholesterol biosynthesis in *Eriocheir sinensis* by modulating SREBP‐1 and MTTP expression, but excessive intake may impair lipid metabolism due to hepatotoxicity [16]. While in tilapia, it regulated adipose tissue metabolism via cytokine‐mediated (IL‐1β/TNF‐α) modulation of lipoprotein lipase activity [17]. High‐niacin‐fed fish showed no hepatic pathological alterations, with a tendency toward reduced lipid accumulation. Thus, the increased expression of IL‐1β and TNF‐α mRNA appears to be linked to changes in lipid metabolism rather than an inflammatory response [17, 18]. Most notably, recent work has identified the SIRT1/GLUT1/HK signaling axis as a conserved mechanism for niacin’s regulation of glycolytic, TCA cycle, and gluconeogenic pathways in tilapia [19]. While mammalian niacin metabolism is well‐characterized, aquatic species research remains in a nascent stage. Given the fundamental physiological differences between aquatic and terrestrial vertebrates [20], systematic investigation of niacin’s metabolic regulation in aquatic models holds both theoretical significance for comparative nutrition and practical implications for aquaculture innovation.

The Nile tilapia (*Oreochromis niloticus*) is an ideal experimental model organism because it has a fast growth rate, high reproductive efficiency, and significant commercial value [11]. Notably, tilapia demonstrates particular sensitivity to niacin’s physiological effects, establishing it as a preferred model for aquatic niacin research [11, 21]. Capitalizing on these advantages, the current study employs Nile tilapia to characterize niacin’s effects on glucose/lipid metabolism and liver health. Our findings would provide a systematic framework for optimizing niacin utilization in sustainable aquaculture practices.

## 2. Materials and Methods

### 2.1. Experimental Animals and Diets

Juvenile Nile tilapia were obtained from Guangzhou Tianfa Aquatic Technology Co., Ltd. (Guangzhou, China). Prior to the experiment, all fish were acclimatized for 2 weeks in rearing systems while being fed a commercial diet. For the formal feeding trial, healthy fish with an initial average body weight (4.5 ± 0.5 g) were randomly allocated into 12 tanks (160‐L tank, 25 fish per tank, three replicates per treatment group). The fish were fed the experimental diets twice daily (9:30 and 16:30) at the rate of 4% of body weight for 8 weeks under the following conditions: Water temperature and dissolved oxygen levels were remained at 26°C–31°C and above 5.0 mg/L, respectively, while total ammonia‐nitrogen was kept below 0.2 mg/L. Animal experiments were strictly conducted in adherence to the Guide for the Care and Use of Laboratory Animals approved by the Committee on the Ethics of Animal Experiments of Shantou University, Guangdong, China (Approval ID: 202509003).

Based on the reported basal niacin requirement for tilapia of ~100 mg/kg [4], four experimental diets were formulated. These included a niacin‐free diet (0 mg/kg), diets with moderate niacin supplementation (50 and 100 mg/kg), and a diet with an excessive level (200 mg/kg), using a niacin source of 99% purity (Shanghai Macklin Biochemical Co., Ltd.). The composition of the diets was detailed in Table 1. Among these levels, the 200 mg/kg niacin supplementation exceeded both the basal niacin requirement of juvenile tilapia [21]. The diets used casein and gelatin as the primary protein sources, corn starch as the main carbohydrate source, and soybean oil as the dietary lipid source. The dry ingredients were first ground into fine powder, then thoroughly mixed with precisely measured amounts of oil and water before being processed into 2‐mm diameter pellets using a laboratory pellet extruder.

**Table 1:** Formulations and proximate compositions of the experimental diets.

Ingredients (g)	0‐NA	50‐NA	100‐NA	200‐NA
Casein	320	320	320	320
Gelatin	110	110	110	110
Corn starch	450	450	450	450
Soybean oil	60	60	60	60
Vitamin (without niacin)^a^	15	15	15	15
Minerals^b^	20	20	20	20
Cellulose	9.75	9.70	9.65	9.55
Choline chloride	5	5	5	5
Ca(H_2_PO_4_)_2_	10	10	10	10
Dimethyl‐β‐propiothetin	0.25	0.25	0.25	0.25
Niacin	0	0.05	0.1	0.2
Total	1000	1000	1000	1000
Proximate composition (%, dry matter)				
Dry matter	89.6	91.1	90.7	90.2
Crude protein	39.4	38.8	39.1	39.5
Crude lipid	6.0	6.2	6.4	5.9
Ash	7.1	6.7	6.5	6.9
Niacin	0.0006	0.041	0.088	0.18

^a^Vitamin premix, (mg or IU/kg, international units): 5000,000 IU Vitamin A; 1000,000 IU Vitamin D3; 15,000 mg Vitamin E; 4000 mg Vitamin K3; 8000 mg Vitamin B1; 10,000 mg Vitamin B2; 10,000 mg Vitamin B6; 20 mg Vitamin B12; 60,000 mg Inositol; 20,000 mg Pantothenic acid; 1000 mg Folic acid; 100 mg Biotin; 50,000 mg Vitamin C.

^b^Mineral premix, (g/kg): 314.0 g CaCO_3_; 469.3 KH_2_PO_4_; 147.4 g MgSO_4_·7H_2_O; 49.8 g NaCl; 10.9 g Fe(II) gluconate; 3.12 g MnSO_4_·H_2_O; 4.67 g ZnSO_4_·7H_2_O; 0.62 g CuSO_4_·5H_2_O; 0.16 g KI; 0.08 g CoCl_2_·6H_2_O; 0.06 g NH_4_ molybdate; 0.02 g NaSeO_3_.

### 2.2. Sampling and Growth Performance Analysis

After the 8‐week feeding trial, the fish were starved overnight and subsequently anesthetized by immersion in a buffered solution of tricaine methanesulfonate (MS‐222; Sigma–Aldrich, USA) at a concentration of 200 mg/L. Then, the number and total weight of the fish in each group were recorded. 12 fish (four fish from each tank) per dietary group were randomly selected for body length and weight measurements. Blood samples collected from the caudal vein was left at 4°C overnight followed by centrifugation at 3000 rpm for 10 min, and the serum was stored at −80°C for biochemical assays. After dissection, the visceral mass, liver, and mesenteric fat were weighed to calculate the viscerosomatic index (VSI), hepatosomatic index (HSI), mesenteric fat index (MFI), and condition factor (CF). Liver tissues (nine fish per treatment) were collected into two tubes for biochemical and molecular analyses, and immediately frozen in liquid nitrogen. The samples for different indicator analyses were collected from the same set of individuals.

### 2.3. Biochemical Analysis

Proximate composition analysis of the experimental diets was performed in accordance with Association of Official Analytical Chemists (AOAC) guidelines. Serum insulin was measured using a fish‐specific ELISA kit (HB407‐QT; Shanghai Hengyuan Biotechnology Co., Ltd.). The activities of aspartate aminotransferase (AST), alanine aminotransferase (ALT), superoxide dismutase (SOD; A001‐3‐2), and catalase (CAT; A007‐1‐1) were determined with commercial kits manufactured by Nanjing Jiancheng Bioengineering Institute. Additionally, the contents of TG (A110‐1‐1), malondialdehyde (MDA, A003‐1‐2), glucose (GLU, A154‐1‐1), and pyruvate (A081‐1‐1) were also detected with commercial kits (Nanjing Jiancheng Bioengineering Institute). NAD^+^/NADH (S0176S) and NADP^+^/NADPH (S0180S) were analyzed with commercial kits (Beyotime Biotechnology). All assays strictly followed the manufacturers’ guidelines to ensure accuracy and reproducibility.

### 2.4. Histological Analysis

Liver tissue samples (three per dietary treatment) were fixed in 4% paraformaldehyde (PFA) for 48 h, followed by gradient ethanol dehydration (70%→80%→90%→100%) and xylene clearing, then embedded in paraffin blocks. Sections of 5 μm thickness were cut using a microtome and mounted on glass slides. For staining, nuclei were dyed blue with hematoxylin for 5–10 min, differentiated in 1% acid alcohol, and blued in running water, while cytoplasm was counterstained pink with eosin for 1–3 min. Finally, slides were dehydrated through an ethanol gradient, cleared in xylene, and permanently mounted with neutral balsam for long‐term preservation. For each sample, 5–10 non‐overlapping fields were randomly examined under a light microscope at magnifications of ×400. Hepatocytes containing lipid vacuoles and the total number of hepatocytes were counted in each field. Vacuoles were carefully distinguished by size and morphology, excluding artifacts like nuclear displacement or staining irregularities. The degree of vacuolation was quantified as the percentage of vacuolated hepatocytes: (number of vacuolated hepatocytes/total hepatocytes) × 100%.

### 2.5. RNA Extraction and qPCR Analysis

Total RNA was extracted from nine samples per treatment using TRIzol reagent (Vazyme Biotech) and quantified via NanoDrop 2000 spectrophotometry. For cDNA synthesis, total RNA (1 μg) was reverse‐transcribed using ABScript Neo RT Master Mix (ABclonal) with integrated gDNA removal. Quantitative PCR was performed on an ABI 7300 system with BrightCycle Universal SYBR Green qPCR Mix (ABclonal) following the Ultra SYBR protocol (No. PC3302; Aidlab, China). Relative gene expression was calculated via the comparative threshold cycle (ΔΔCt) method, normalized to reference genes *β-actin* and *ef1*α (primer sequences in Table 2). The specificity of all primers used in the analysis was verified by melt curve analysis following amplification. All procedures strictly adhered to manufacturer protocols.

**Table 2:** Primer pair sequences of the genes used for real‐time PCR (qPCR).

Gene	Forward primer (5′‐to 3′‐)	Reverse primer (5′‐to 3′‐)	Amplification efficiency (%)	Accession No.
*β-actin*	AGCCTTCCTTCCTTGGTATGGAAT	TGTTGGCGTACAGGTCCTTACG	97	KJ126772.1
*ef1a*	ATCAAGAAGATCGGCTACAACCCT	ATCCCTTGAACCAGCTCATCTTGT	99	KJ123689.1
*fasn*	TCATCCAGCAGTTCACTGGCATT	TGATTAGGTCCACGGCCACA	103	GU433188
*accα*	TAGCTGAAGAGGAGGGTGCAAGA	AACCTCTGGATTGGCTTGAACA	95	XM_005471970
*acly*	AAAAGCTTTGATGAGCTTGGGG	TACAGTGGGAGGAGGCAACTCTT	95	XM_003442027
*dgat*	GCTTGAATTCTGTCACCCTGAAGA	ACCTGCTTGTAGGCGTCGTTCT	94	XM_003458972
*atgl*	GACACATGCTGCAAAGCACT	ACCAGGACGTTTTCTCCGTC	99	XM_003440346.5
*hsl*	AGTTCACTCCAGCCATTCGG	TGGCTGCTACCCCTATTCCT	101	XM_005463937.4
*mgl*	GGGCTCCATCGAGTCCAAAT	AATGATACTCGCATCCCGCC	93	XM_005478351.4
*lc3a*	GTCCAGCAGATCCGTGAGC	TGCCAGGAACTTGGTCTTGTC	95	XM_019356796.2
*lc3b*	GCCTCCAGCTAAACTCCAACC	CGCTCTCGCTCGTACACCTC	104	XM_003439438.5
*atg12*	TCATATCTCGCTTCCTCAAGC	CCCTACTTCTTGATCCGGTGA	98	XM_025911479.1
*cpt-1 b*	AAGGGACGTTACTTCAAGGTG	TCCGACTTGTCTGCCAAGAT	95	GQ395696
*aco*	AGTCCCACTGTGAGCTCCATCAA	CAGACCATGGCAGTTTCCAAGA	97	KF918710
*pparα*	GTTCCTCAAGAGTCTCCGCC	AAAGAGCTAGGTCGCTGTCG	103	KF871430
*glut1*	TCTTCATCCCCGCTGTGATACA	TTCTTCAGCACTGATTTGGCCTT	101	FJ914657
*glut2*	CATTGGCATTCTAATCAGCCAGGT	TTGTAATATTGCTGGCGCTCCA	94	XM_003442884.5
*glut4*	GCAGGAGGAAAGCCATGCTTATA	ATCATTTCAAAGGAGCGGCAGA	98	XM_003458705.4
*ir*	TTCAGCTGCCACCACGT	TCATCAGCTCCATCACCACCA	104	KC517071.1
*g6pase*	AGACCTTATTGGTGGGTTCACGA	CTGAAGGACTTCCTGGTCCAGTTT	101	XM_003448671.4
*fbpase*	ACCGGACAATAGCGGAAAATACA	TGGCGAATATTGTTCCTATGGAGA	92	XM_003449650.4
*pepck*	TGGAAGAACAAACCTTGGCG	TGGGTCAATAATGGGACACTGTCT	99	XM_003448375
*gs*	CCTCACTCTGCGCTGTTATTC	CAGCGGCATGCCTTCAGTTT	95	XM_013276796.3
*gp*	AGCGCAAGCAGATCAGTATCA	CCCTTGGCGTTGCAATGTTT	102	XM_003442862.4
*gk*	GACATGAGGACATTGACAAGGGAA	CTTGATGGCGTCTCTGAGTAAACC	93	XM_003451020.2
*hk*	CACTGAGCTCAAGGATGACCA	CTCGGGCGTGTCGTAGATTT	104	XM_003454508
*pk*	CAGCATAATCTGCACCATCGGT	ATGAGAGAAGTTAAGACGGGCGA	95	XM_005472621.3
*cs*	AGCACCACAGTTTACCAG	AGTGTTGACAAACCCAGA	99	XM_003438897
*idh*	ACGCATCGCTGAGTACGCCTT	AGACCGTCTGACATCCGCATGA	105	XM_003437590.5
*sod1*	GCCCACACTTCAATCCCTACAA	GGCTCTCTTCATTTCCTCCTTT	93	XM_003446807.4
*gsh-px1*	ACCTTCATTCTCGCTACTCC	GCAGTTCTCCTGATGTCCAA	96	NM_001279711.1
*gst*	TAATGGGAGAGGGAAGATGG	CTCTGCGATGTAATTCAGG	98	NM_001279635.1

*Note: acca*, acetyl‐CoA carboxylase alpha; *aco*, acyl‐CoA oxidase; *acly*, ATP‐citrate lyase; *atg12*, autophagy related 12; *atgl*, adipose triglyceride lipase; *cpt-1b*, carnitine palmitoyltransferase 1b; *dgat*, diacylglycerol acyltransferase; *EF1α*, eukaryotic translation elongation factor 1 alpha; *fasn*, fatty acid synthase; *fbpase*, fructose‐1,6‐bisphosphatase 1; *g6pase*, glucose‐6‐phosphatase; *glut1*, glucose transporter 1; *glut2*, glucose transporter 2; *glut4*, glucose transporter 4; *gsh-px1*, glutathione peroxidase 1; *gst*, glutathione S‐transferase; *lc3a*, microtubule‐associated protein 1 light chain 3 alpha; *lc3b*, microtubule‐associated protein 1 light chain 3 beta; *mgl*, monoglyceride lipase; *pepck*, phosphoenolpyruvate carboxykinase; *pparα*, peroxisome proliferator‐activated receptor alpha; *sod1*, superoxide dismutase 1.

Abbreviations: cs, citrate synthase; gk, glucokinase; gp, glycogen phosphorylase; gs, glycogen synthase; hk, hexokinase; hsl, hormone‐sensitive lipase; idh, isocitrate dehydrogenase; ir, insulin receptor; pk, pyruvate kinase.

### 2.6. Metabolomic Analysis

Liver tissue samples (five per dietary treatment) from tilapia fed C, C + 100 and C + 200 groups were homogenized with 200 μL of pre‐chilled water and two steel beads after flash‐freezing in liquid nitrogen, using a grinder at 55 Hz for two 60‐s cycles. For metabolite extraction, 800 μL of methanol–acetonitrile (1:1, v/v) was introduced, followed by 30‐min ultrasonication and 30‐min incubation at −20°C. After centrifugation (12,000 rpm, 4°C, 10 min), the supernatant was concentrated, dried, and reconstituted in 150 μL of 50% methanol containing 5 ppm 2‐chlorophenylalanine as an internal standard, then filtered through a 0.22‐μm membrane. Quality control (QC) samples were prepared by pooling equal volumes from each sample.

LC‐MS analysis was conducted using an ACQUITY UPLC HSS T3 column (100 Å, 1.8 µm, 2.1 mm × 100 mm), operating at 0.4 mL/min flow rate with column temperature set to 40°C and autosampler maintained at 8°C (injection volume: 2 μL). The mass spectrometric analysis was performed on a Thermo Orbitrap Exploris 120 mass spectrometer controlled by Xcalibur software (Thermo), acquiring data‐dependent acquisition (DDA) spectra in both positive and negative ion modes. Raw data were imported into Compound Discoverer 3.3 (v3.3.2.31, Thermo) for peak alignment, gap‐filling (using the FillGaps algorithm), and normalization (based on total peak area summation). The raw sequencing data have been deposited in a publicly available database MetaboLights as MTBLS13383.

### 2.7. Statistical Analysis

Following verification of homogeneity of variance using Levene’s test, a one‐way ANOVA was performed to compare differences among the groups. Data visualization was conducted using GraphPad Prism 7.0. Results were shown as means ± standard deviation with *n* value as indicated.

## 3. Results

### 3.1. Niacin Deficiency Led to Growth Inhibition, While Excessive Niacin Intake Resulted in Hepatic Lipid Deposition

Compared with the niacin‐free group, dietary niacin supplementation (50–200 mg/kg) significantly promoted the growth of tilapia (Figure 1A). Moreover, the VSI was significantly lower in groups supplemented with 50–100 mg/kg niacin (Figure 1C). However, excessive niacin supplementation (200 mg/kg) resulted in significantly higher condition factor (CF) and VSI compared to other groups (0–100 mg/kg; Figure 1B,D). Additionally, hepatic TG content was significantly elevated in the excessive niacin group (200 mg/kg) compared to other groups (0–100 mg/kg; Figure 1E). Notably, serum TG levels in fish fed 100 mg/kg niacin were significantly lower than in all other groups (Figure 1F). Correspondingly, hepatic vacuolization was more obvious in the excessive niacin group (Figure 1G). In summary, niacin deficiency leads to growth inhibition, while excessive niacin intake results in hepatic lipid deposition.

Figure 1Effect of dietary niacin on growth performance and triglyceride content of tilapia. (A) Body weight (*n* = 12); (B–D) condition factor (CF), viscerosomatic index (VSI), and mesenteric fat index (MFI, *n* = 12); (E,F) triglyceride contents in liver and serum (TG, *n* = 9); (G) hematoxylin‐eosin staining of liver, scale bars, 200 μm (10x) and 50 μm (40x; *n* = 3); Values are presented as mean ± standard deviation. Means with different superscript letters in each column indicate significant differences among dietary treatments; 0‐NA: niacin‐free diet; 50‐NA: 50 mg/kg niacin inclusion diet; 100‐NA: 100 mg/kg niacin inclusion diet; 200‐NA: 200 mg/kg niacin inclusion diet.

**Figure:**
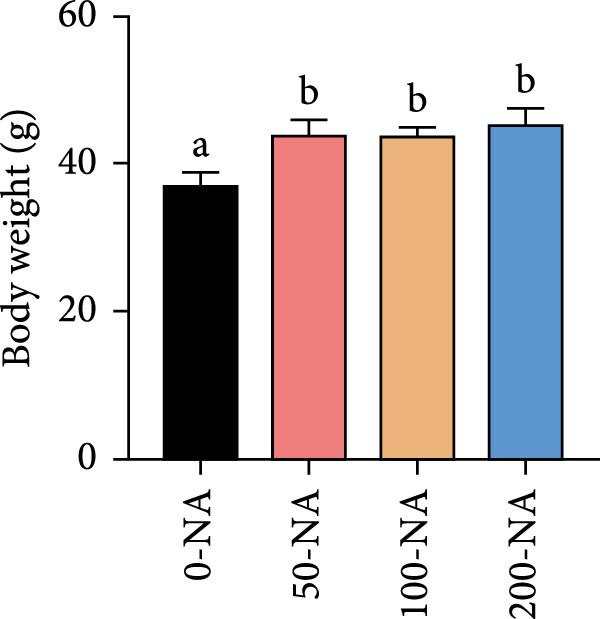
(A)

**Figure:**
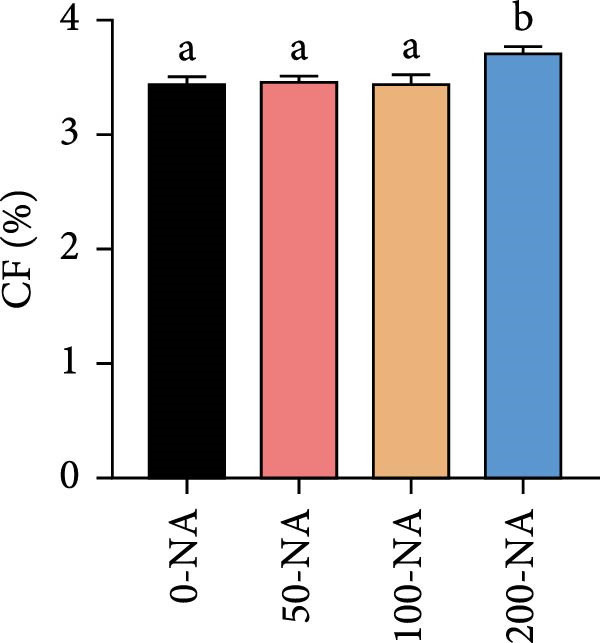
(B)

**Figure:**
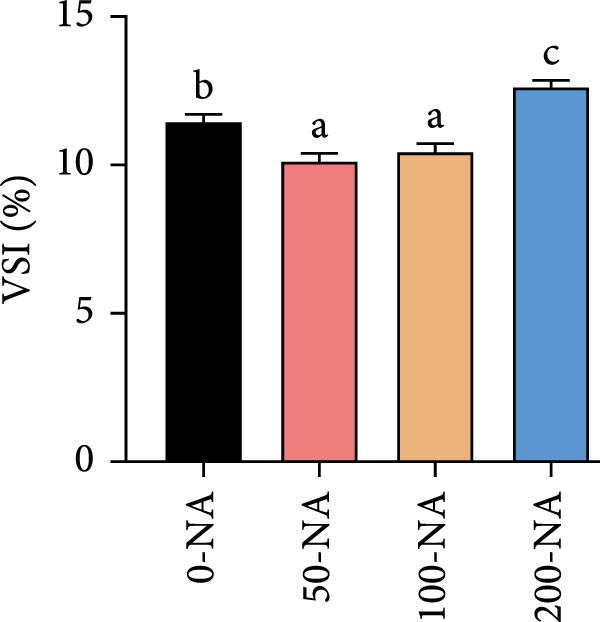
(C)

**Figure:**
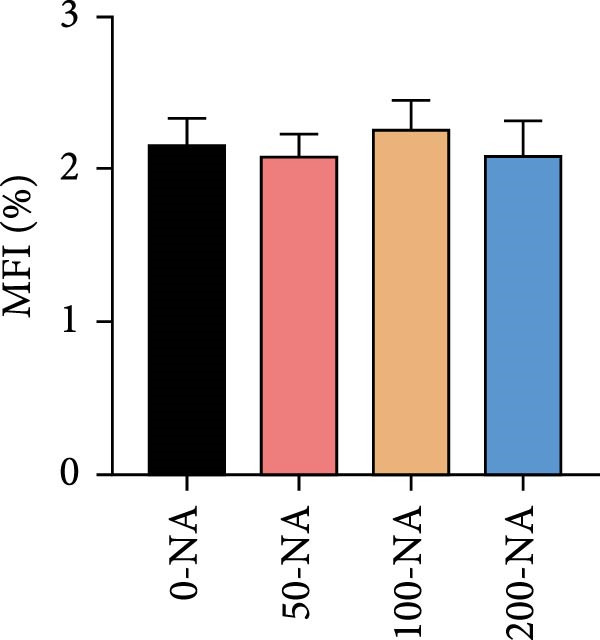
(D)

**Figure:**
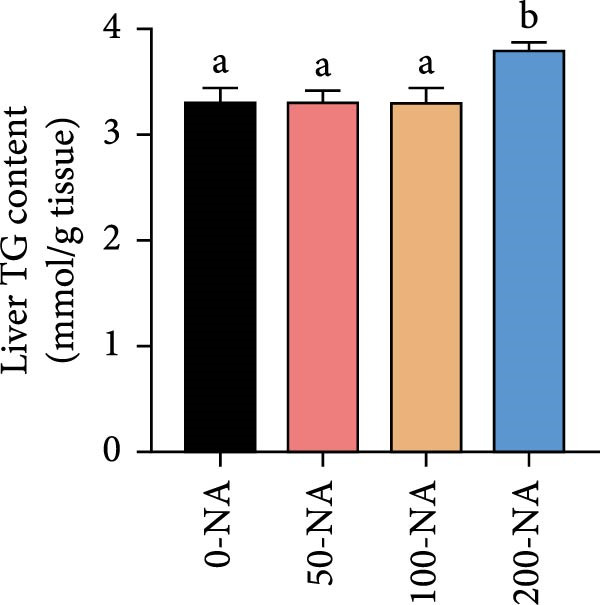
(E)

**Figure:**
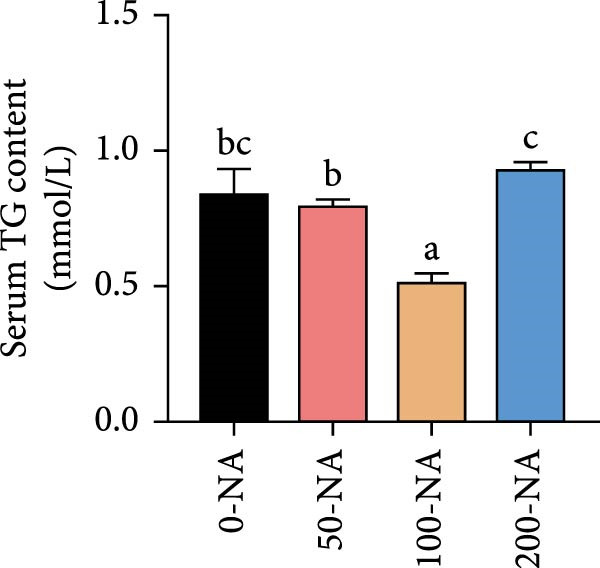
(F)

**Figure:**
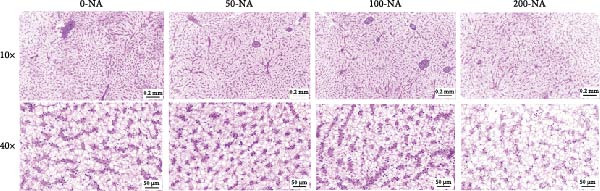
(G)

### 3.2. Excessive Niacin Caused Lipid Accumulation by Enhancing Lipogenesis and Inhibiting Lipid Catabolism

Compared to other groups, the moderate niacin supplementation group (100 mg/kg) downregulated the expression of hepatic lipogenesis‐related genes (*fasn* and *accɑ*) while upregulating genes associated with lipolysis (*atgl*), autophagy (*lc3b*) and fatty acid β‐oxidation (*cpt-1 b* and *pparɑ*; Figure 2A–D). In contrast, excessive niacin supplementation (200 mg/kg) increased the expression of lipogenic genes (*fasn*, *accɑ* and *acly*) while suppressing lipolysis (*atgl*) and autophagy (*lc3a*, *lc3b*; Figure 2A–D). These findings demonstrated that optimal niacin supplementation (100 mg/kg) enhanced lipid catabolism in tilapia, whereas excessive niacin caused lipid accumulation by enhancing lipogenesis and inhibiting lipid catabolism.

Figure 2Effect of dietary niacin on lipid metabolism of tilapia. (A,B) Gene expression levels related to lipid synthesis and hydrolysis (*n* = 9); (C) gene expression levels related to cellular autophagy (*n* = 9); (D) gene expression levels related to fatty acid β‐oxidation (*n* = 9); Values are presented as mean ± standard deviation. Means with different superscript letters in each column indicate significant differences among dietary treatments; 0‐NA: niacin‐free diet; 50‐NA: 50 mg/kg niacin inclusion diet; 100‐NA: 100 mg/kg niacin inclusion diet; 200‐NA: 200 mg/kg niacin inclusion diet.

**Figure:**
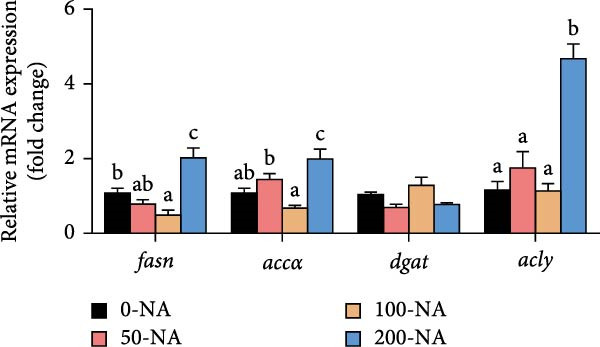
(A)

**Figure:**
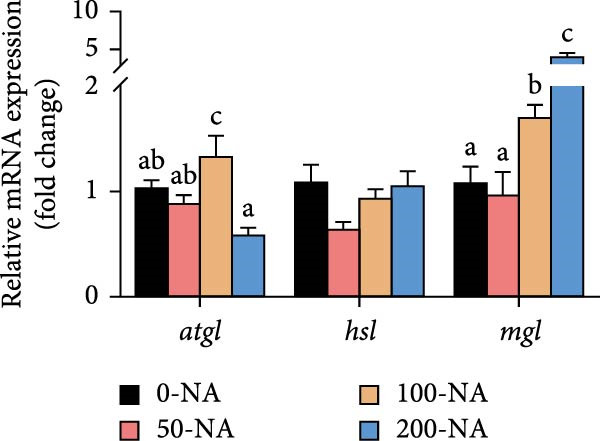
(B)

**Figure:**
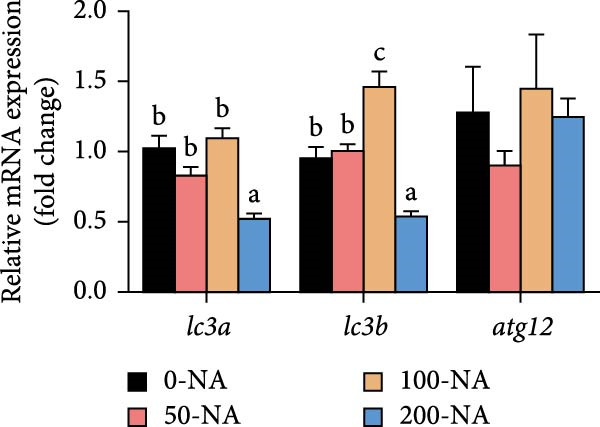
(C)

**Figure:**
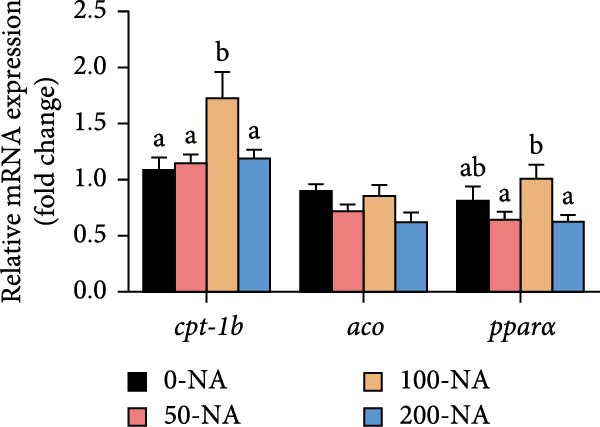
(D)

### 3.3. Optimal Niacin Supplementation Maintained Glucose Metabolic Homeostasis, Whereas Excessive Niacin Inhibited Glucose Oxidation

Compared to other groups, the niacin‐free group exhibited significantly lower serum glucose levels (Figure 3A). Tilapia fed with moderate niacin supplementation (100 mg/kg) exhibited significantly higher serum insulin levels compared to the niacin‐free group, while hepatic pyruvate content gradually increased with niacin supplementation (Figure 3B,C). Furthermore, compared to the niacin‐free or excessive‐supplementation groups, the moderate niacin group significantly upregulated the expression of genes related to glucose transport (*glut1*) and the TCA cycle (*cs* and *idh*; Figure 3D,G). Moreover, excessive niacin supplementation (200 mg/kg) significantly suppressed hepatic glycogen synthesis (*gs*) and TCA cycle (*cs* and *idh*)‐related gene expression while promoting genes associated with glycogenolysis (*gp*), glycolysis (*gk*), and gluconeogenesis (*pepck*; Figure 3E–G). These results indicated that optimal niacin supplementation maintains glucose metabolic homeostasis in tilapia liver, whereas excessive niacin inhibited glucose oxidation for energy production.

Figure 3Effect of dietary niacin on glucose metabolism of tilapia. (A) Serum glucose (*n* = 9); (B) serum insulin (*n* = 9); (C) liver pyruvate content (*n* = 9); (D) gene expression levels related to glucose uptake (*n* = 9); (E,F) gene expression levels related to gluconeogenesis and glycolysis (*n* = 9); (G) gene expression levels related to the tricarboxylic acid cycle (*n* = 9); Values are presented as mean ± standard deviation. Means with different superscript letters in each column indicate significant differences among dietary treatments; 0‐NA: niacin‐free diet; 50‐NA: 50 mg/kg niacin inclusion diet; 100‐NA: 100 mg/kg niacin inclusion diet; 200‐NA: 200 mg/kg niacin inclusion diet.

**Figure:**
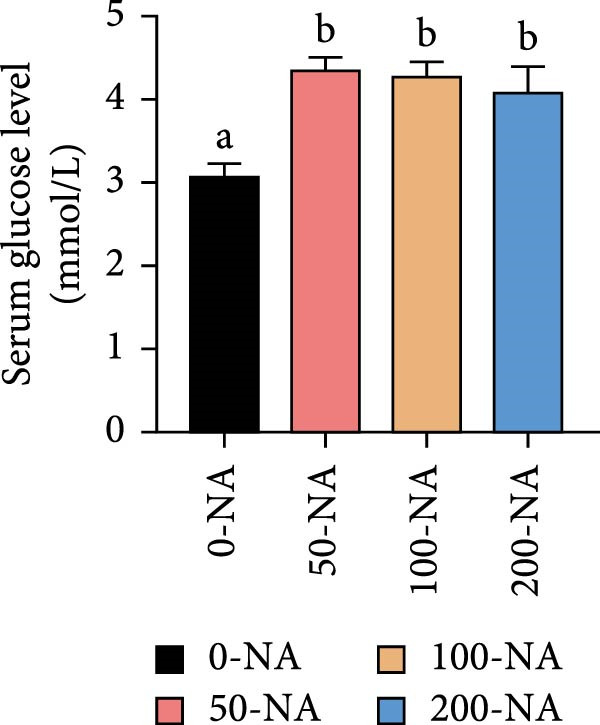
(A)

**Figure:**
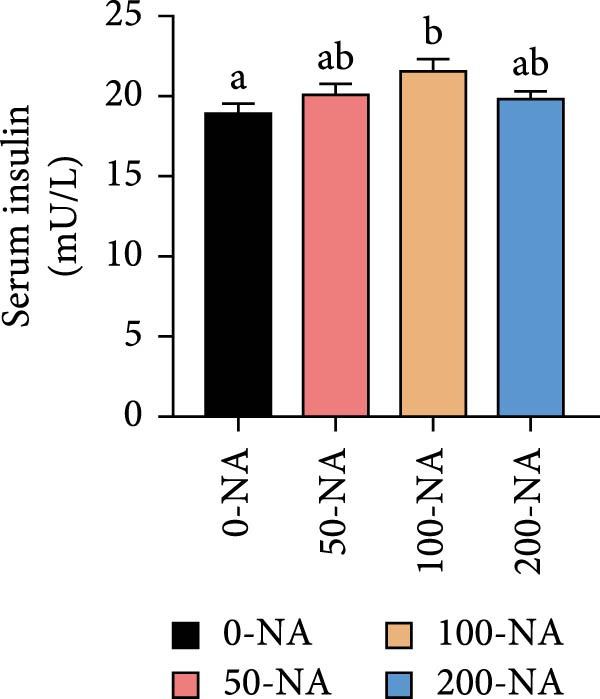
(B)

**Figure:**
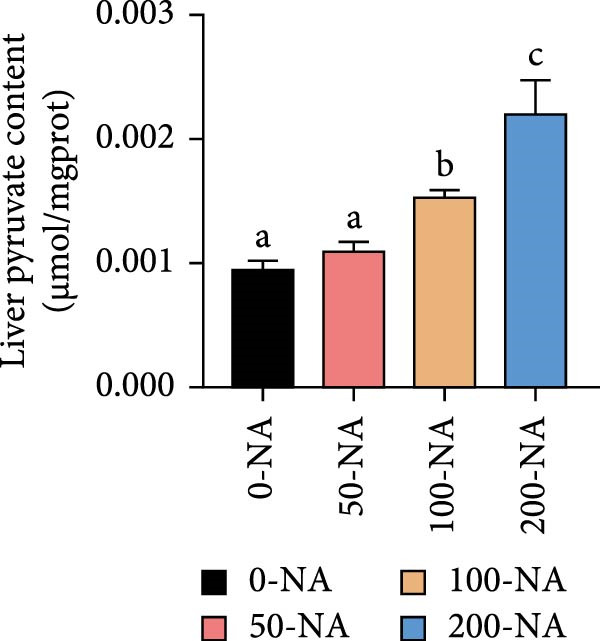
(C)

**Figure:**
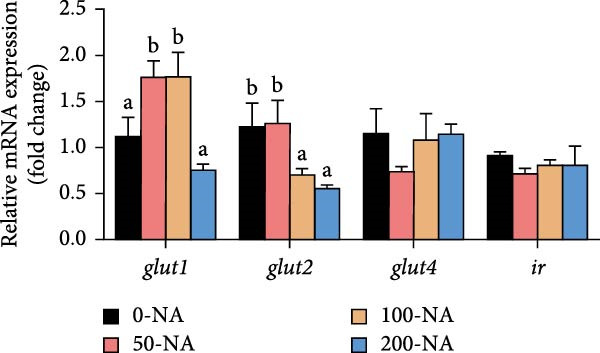
(D)

**Figure:**
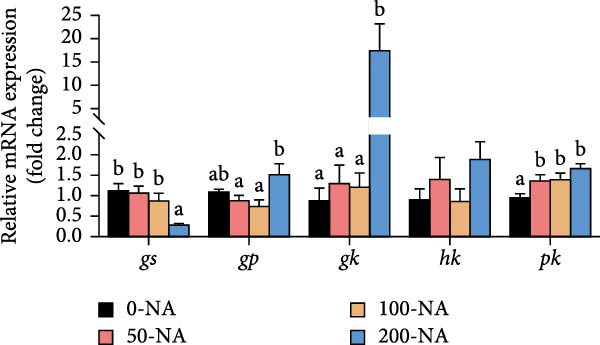
(E)

**Figure:**
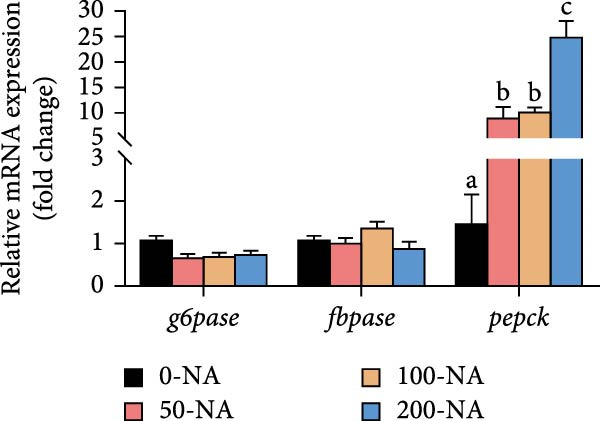
(F)

**Figure:**
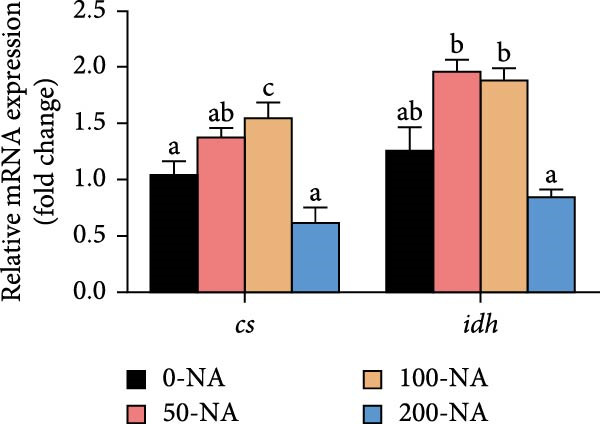
(G)

### 3.4. Excessive Niacin Supplementation Increased Liver Oxaloacetate Content

Metabolomic PCA analysis revealed clear separation among the 0‐NA, 100‐NA (100 mg/kg niacin) and 200‐NA (200 mg/kg niacin) groups (Figure 4A,B), demonstrating that niacin dosage significantly alters the metabolic profile of tilapia. Pathway enrichment analysis indicated that both niacin deficiency and excess groups exhibited significant enrichment in pathways related to *metabolic processes* (e.g., amino acid, carbohydrate, and lipid metabolism) and *environmental response*, suggesting that niacin deficiency and over‐dose primarily disrupts energy metabolism pathways (Figure 4C–F). Further analysis of differential metabolites showed that niacin deficiency increased aspartate, valine, serine, and pantothenate levels, while decreasing oxaloacetate and glutathione (Figure 4G). However, excessive niacin caused accumulation of oxaloacetate, aspartate, and glutathione, but reduced glutamate content (Figure 4G). Additionally, liver NAD^+^ and NADP^+^ contents in Nile tilapia increased with increasing dietary niacin levels (Figure 4I,J). These results suggested that oxaloacetate accumulated in the TCA cycle was converted to aspartate.

Figure 4Effect of dietary niacin on metabolomics of tilapia. (A) PCA score plot; (B) heatmap showing the clustering of differential metabolites across samples; (C) bubble plot of KEGG pathway enrichment; (D,F) volcano plot of differential metabolites; (E,G) bar plot of KEGG pathway enrichment; (H) heatmap of differential metabolites; (I,J) liver NAD^+^ and NADP^+^ content (*n* = 9). Means with different superscript letters in each column indicate significant differences among dietary treatments; 0‐NA: niacin‐free diet; 100‐NA: 100 mg/kg niacin inclusion diet; 200‐NA: 200 mg/kg niacin inclusion diet.

**Figure:**
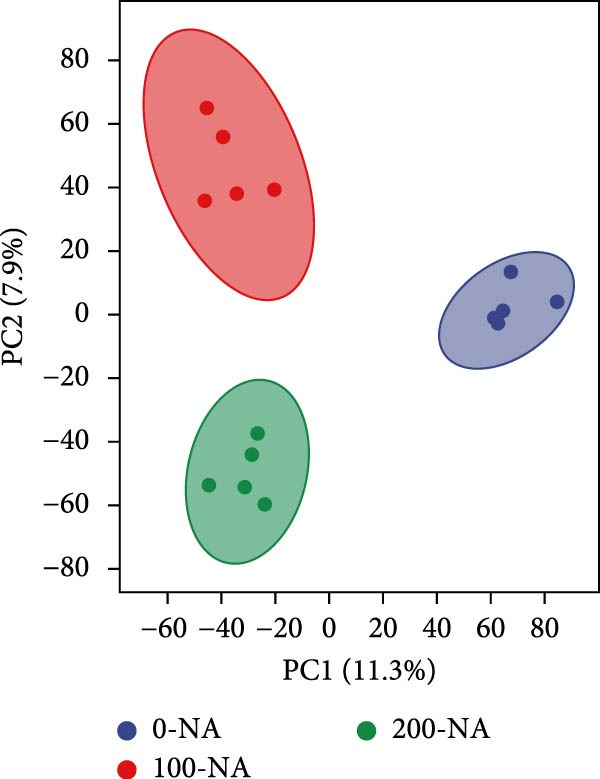
(A)

**Figure:**
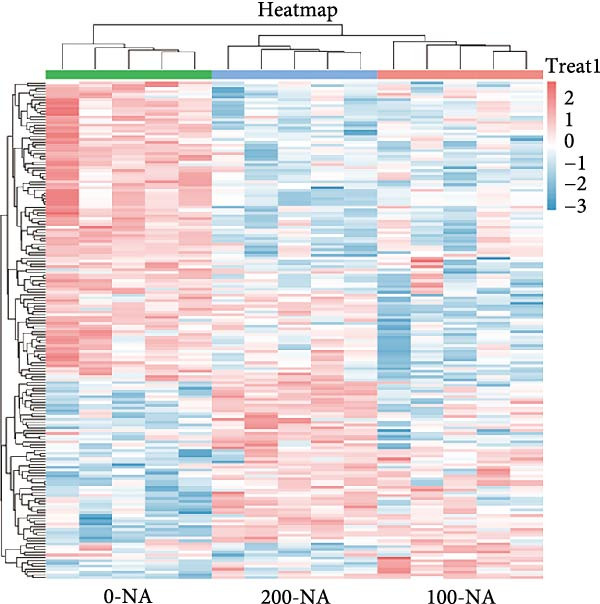
(B)

**Figure:**
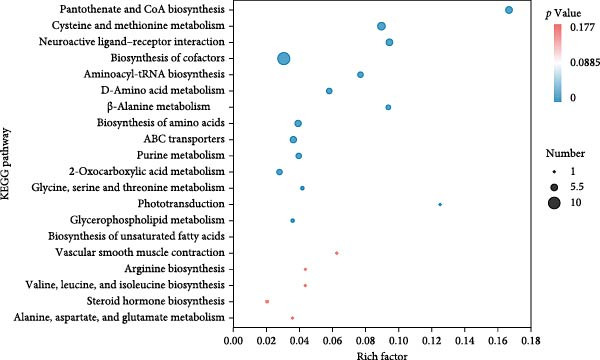
(C)

**Figure:**
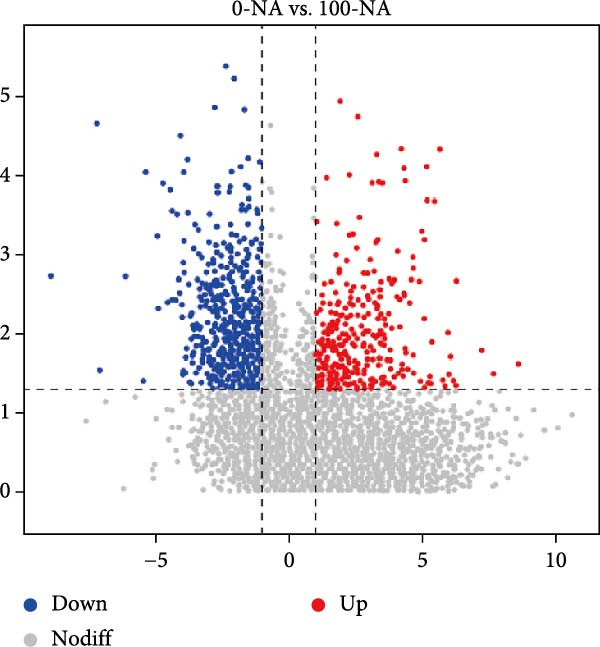
(D)

**Figure:**
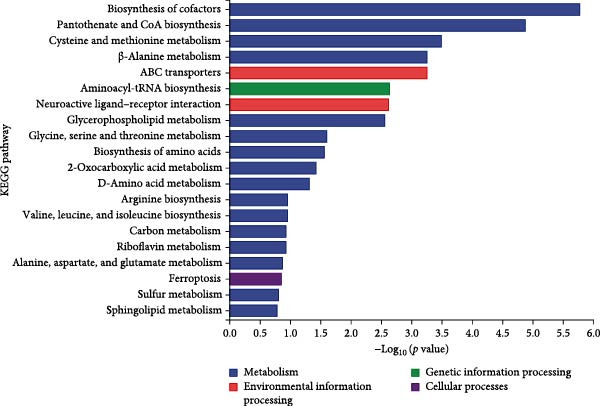
(E)

**Figure:**
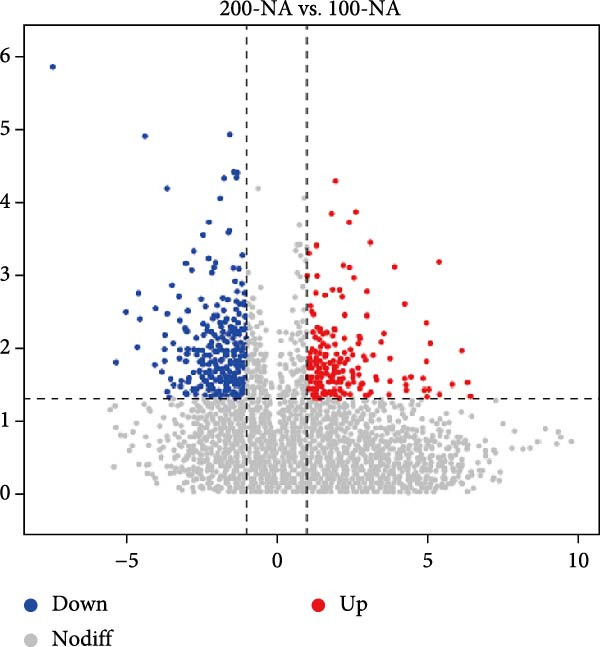
(F)

**Figure:**
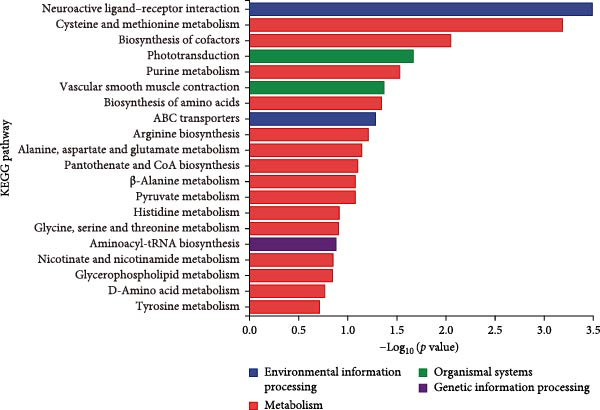
(G)

**Figure:**
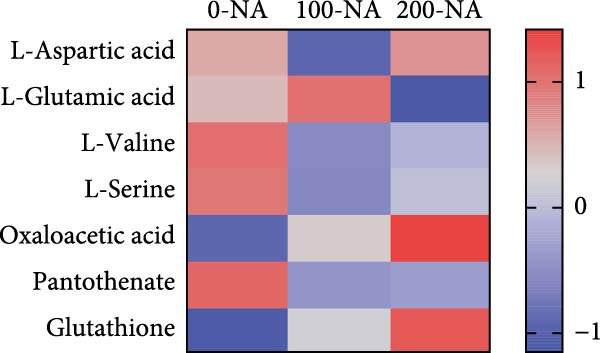
(H)

**Figure:**
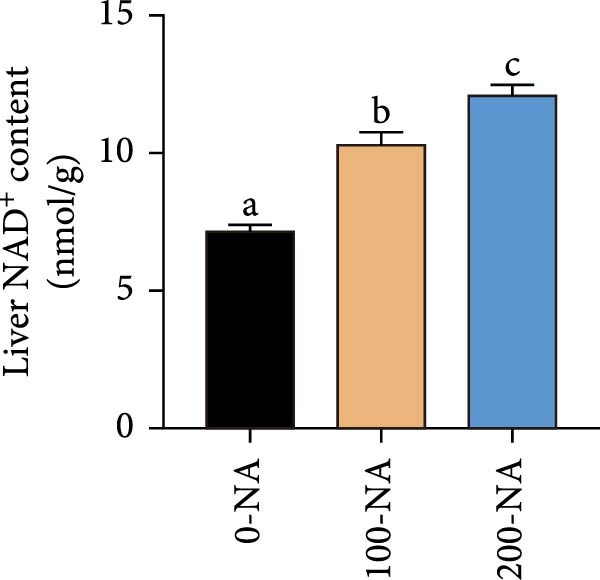
(I)

**Figure:**
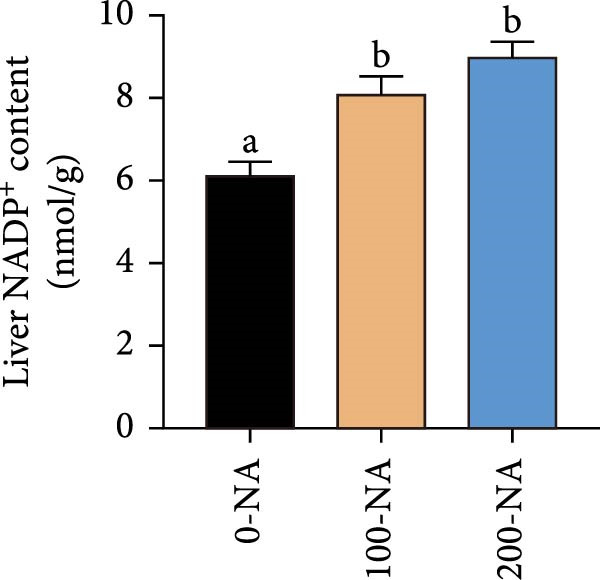
(J)

### 3.5. Optimal Niacin Supplementation Improved Liver Health and Antioxidant Capacity (AOC)

Tilapia fed with moderate niacin supplementation (50–100 mg/kg) exhibited significantly lower serum ALT enzyme activity compared to both the niacin deficiency and excess groups (Figure 5A). Furthermore, hepatic SOD and CAT activities were significantly elevated in this both groups relative to other treatments (Figure 5B,C). Molecular analysis revealed significant upregulation of the oxidative stress‐related gene (glutathione S‐transferase*; gst*) in the moderate supplementation group (Figure 5D). Therefore, optimal niacin supplementation improved liver health and AOC.

Figure 5Effect of dietary niacin on liver health and antioxidant capacity of tilapia. (A) Serum alanine aminotransferase (ALT) enzyme activity (*n* = 9); (B) hepatic superoxide dismutase (SOD) enzyme activity (*n* = 9); (C) hepatic catalase (CAT) enzyme activity (*n* = 9); (D) gene expression levels related to antioxidant activity (*n* = 9). Means with different superscript letters in each column indicate significant differences among dietary treatments; 0‐NA: niacin‐free diet; 50‐NA: 50 mg/kg niacin inclusion diet; 100‐NA: 100 mg/kg niacin inclusion diet; 200‐NA: 200 mg/kg niacin inclusion diet.

**Figure:**
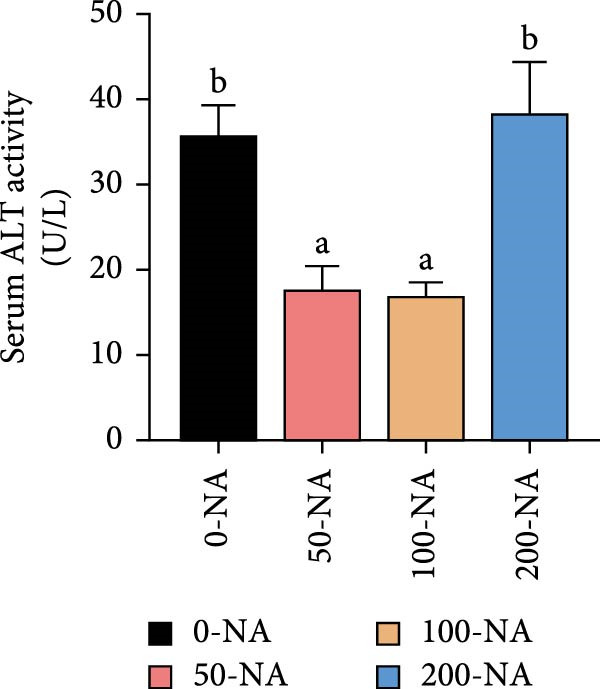
(A)

**Figure:**
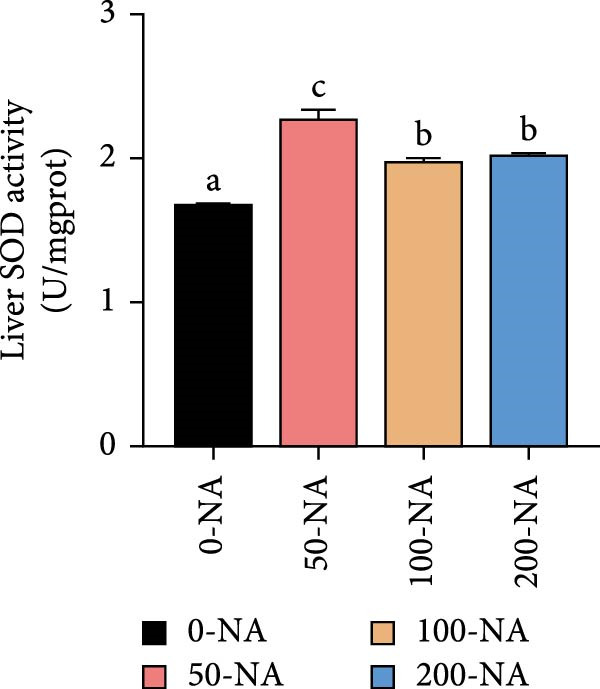
(B)

**Figure:**
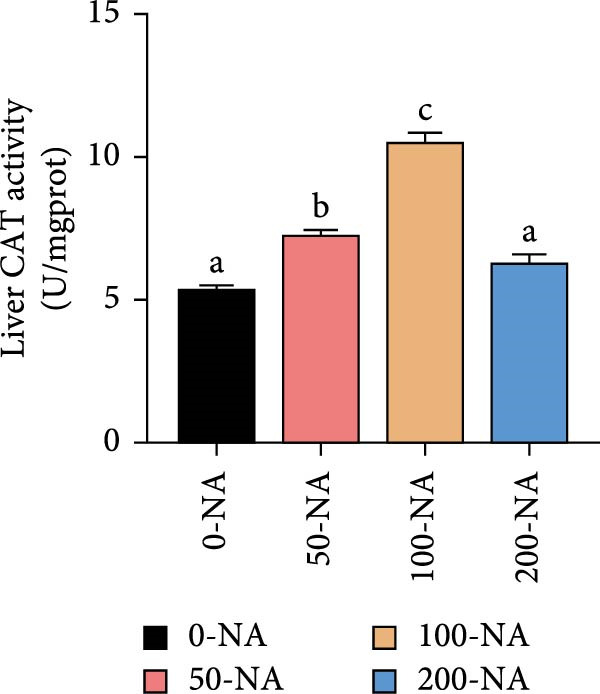
(C)

**Figure:**
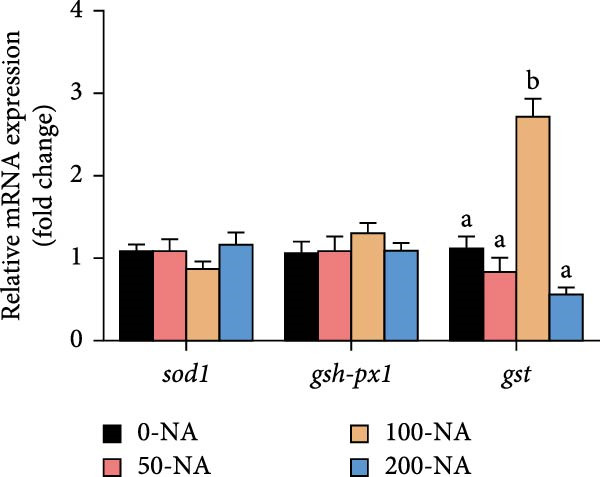
(D)

## 4. Discussion

### 4.1. Niacin Deficiency Led to Growth Inhibition, While Excessive Niacin Intake Resulted in Hepatic Lipid Deposition

Niacin (vitamin B_3_) is an essential nutrient for fish. Deficiency usually leads to various health issues, including anorexia, cutaneous hemorrhage, and branchial edema. Previous studies indicated that proper dietary niacin supplementation improves growth performance in various fish species. For instance, optimal dietary niacin levels for Nile tilapia growth ranged between 20 and 120 mg/kg [4]. Grass carp fed diets containing 9.8–32.2 mg/kg niacin showed significantly improved specific growth rate, weight gain rate and survival rate, alongside a reduced feed conversion ratio [7]. Hybrid sturgeon (*Acipenser baerii ♀* × *A. schrenckii ♂*) exhibited a significantly lower weight gain rate in the non‐supplemented group compared to groups receiving 30–1200 mg/kg niacin; furthermore, the condition factor and VSI were significantly lower in the 120 mg/kg group than in the 0, 30, and 1200 mg/kg groups [22]. Consistent with these findings, the present study demonstrated that dietary niacin supplementation (50–200 mg/kg) significantly promoted growth in Nile tilapia compared to the niacin depleted group. Moreover, fish receiving 50–100 mg/kg niacin also exhibited a significantly reduced VSI. However, excessive supplementation (200 mg/kg) resulted in significantly higher VSI and CF values compared to other groups. Therefore, the effect of niacin on Nile tilapia growth is dose‐dependent: moderate levels (50–100 mg/kg) significantly enhance growth performance, while deficiency or excess might lead to negative outcomes.

Previous studies have demonstrated that niacin participates in the regulation of lipid metabolism biological processes in animals, particularly since the catabolic process of lipid relies on the availability of NAD^+^ [23, 24]. For example, dietary niacin supplementation at 60–120 mg/kg significantly reduced plasma cholesterol, TG, and free fatty acid levels in broiler chickens [25]. However, excessive niacin intake (400–800 mg/kg) significantly increased the crude fat content in the hepatopancreas of Chinese mitten crab (*Eriocheir sinensis*) [26]. In grass, a significant increase in body lipid content was observed in fish fed diets supplemented with 10 mg/kg niacin [7]. In the present study, liver TG content was significantly higher in Nile tilapia receiving excessive niacin (200 mg/kg) compared to other groups (0–100 mg/kg). Corroborating these biochemical findings, excessive niacin supplementation induced pronounced hepatocellular vacuolation, suggesting hepatic lipid metabolic dysregulation leading to TG accumulation.

In mammals, niacin exerts its hypolipidemic effects in the liver by directly suppressing diacylglycerol acyltransferase (DGAT) activity, leading to a reduction in TG synthesis. Furthermore, niacin regulates both lipogenesis and lipolysis in animals through hormonal pathways involving adiponectin, leptin, and insulin, as well as key signaling mediators such as GPR109A, SIRT, and AMPK [27]. However, excessive niacin acts like a hormone‐like signaling molecule by activating specific receptors (GPR109A) on adipocytes, strongly inhibiting the mobilization of lipolysis [27]. In the present study, compared to other groups, tilapia fed with 100 mg/kg niacin exhibited significantly downregulated expression of hepatic genes involved in lipogenesis, alongside upregulated expression of genes related to lipolysis, autophagy and fatty acid β‐oxidation. In contrast, excessive niacin (200 mg/kg) significantly upregulated lipogenic and autophagy, ultimately leading to hepatic lipid accumulation in Nile tilapia. Therefore, moderate niacin supplementation (100 mg/kg) improves lipid metabolism and energy utilization in Nile tilapia. Conversely, excess niacin promoted lipogenesis while inhibiting lipid catabolism, resulting in hepatic steatosis.

### 4.2. Optimal Niacin Supplementation Maintained Glucose Metabolic Homeostasis, Whereas Excessive Niacin Suppressed Mitochondrial TCA Cycle

After digestion and absorption, dietary niacin is converted into NAD^+^ and NADP^+^, which function as essential electron acceptors or hydrogen donors in redox reactions underpinning energy metabolism, including glycolysis, the TCA cycle, and gluconeogenesis [28]. Research on mammals has demonstrated that niacin stimulated amphiregulin (AREG) expression through the SIRT2‐C/EBPβ pathway and activated the PI3K‐AKT signaling pathway, thereby upregulating‐phosphofructo‐2‐ kinase/fructose‐2,6‐bisphosphatase 3(PFKFB3)expression and enhancing glycolysis [29]. Recent studies on Nile tilapia suggested that dietary niacin may enhance glycolysis through the SIRT1/GLUT1 signaling pathway [19]. In the present study, hepatic NAD^+^ and NADP^+^ content in Nile tilapia increased correspondingly with rising dietary niacin levels. Serum glucose levels were significantly lower in the niacin‐free group than in all supplemented groups, which is consistent with previous findings in juvenile carp demonstrating that the glucose content in the group supplemented with 50 mg/kg niacin was markedly lower than that in the groups supplemented with niacin 90–110 mg/kg [8]. Additionally, serum insulin levels were significantly elevated in fish fed the optimal supplementation level (100 mg/kg) compared to other group, while hepatic pyruvate content increased with dietary niacin. Therefore, our study found that niacin deficiency caused hypoglycemia in tilapia, which might be associated with the impairment of the gluconeogenesis pathway. This is evidenced by the fact that while the glycolytic pathway was inhibited, the expression of *pepck*, a key gene for gluconeogenesis, was significantly lower than that in all other groups. Further investigation demonstrated that fish receiving moderate niacin levels exhibited significantly upregulated expression of hepatic genes related to glucose transport, glycolysis, gluconeogenesis and TCA cycle compared to other group. Conversely, excessive niacin supplementation (200 mg/kg) significantly inhibited expression of genes involved in glycogen synthesis and the TCA cycle, while promoting expression of genes related to glycogenolysis, glycolysis, and gluconeogenesis. These results suggested that moderate niacin supplementation maintains hepatic glucose metabolism homeostasis in Nile tilapia, whereas excessive intake suppressed oxidative glucose utilization for energy. Regarding the mechanism by which excessive niacin inhibits the TCA cycle in tilapia liver, further investigation is required.

The TCA cycle, catalyzing the complete oxidation of acetyl‐CoA to CO_2_ and H_2_O while generating energy, serves as the central hub integrating catabolic (e.g., glycolysis, β‐oxidation) and anabolic (e.g., gluconeogenesis, lipogenesis) pathways for carbohydrates, lipids, and amino acids [30]. Pantothenate supports TCA cycle function through CoA synthesis, vital for substrate activation and intermediate metabolism [31]. The present results showed downregulated expression of genes encoding citrate synthase and isocitrate dehydrogenase (*cs*, *idh*; Figure 3G). As the rate‐limiting enzyme catalyzing the condensation of acetyl‐CoA with oxaloacetate to form citrate, citrate synthase’s downregulated expression indicated impaired conversion of oxaloacetate to citrate. Additionally, oxaloacetate was converted to aspartate via transamination—this reaction alleviated the feedback inhibition of excessive oxaloacetate on the TCA cycle [32]. Furthermore, oxaloacetate acted as a key co‐substrate for NAD^+^‐dependent dehydrogenases in the TCA cycle, and its accumulation further confirmed TCA cycle stagnation, as the consumption of oxaloacetate and associated NAD^+^‐dependent reactions were suppressed [33]. In the present study, excessive niacin (200 mg/kg) inhibited mitochondrial TCA cycle activity, resulting in the accumulation of oxaloacetate and subsequently increasing the conversion to aspartate. This result indicated the impairment of TCA leads to the accumulation of oxaloacetate, which may be converted into aspartate to alleviate the stress. Furthermore, the impairment of TCA might disrupt mitochondrial redox homeostasis, and consequently increasing reactive oxygen species generation [34, 35]. In the present study, excessive niacin intake also promoted pantothenate‐derived glutathione synthesis, which may help counteract potential mitochondrial oxidative stress associated with TCA cycle dysfunction. However, no direct correlation analysis was performed in the present study between TCA intermediates (e.g., oxaloacetate, α‐ketoglutarate) and anti‐oxidative markers (e.g., glutathione), so direct mechanistic evidence was lacking. Overall, excessive niacin supplementation induced metabolic disturbances and potentially triggered oxidative stress in Nile tilapia, partially through TCA cycle inhibition.

### 4.3. Optimal Niacin Supplementation Improved Liver Health and AOC

NADPH, a crucial cellular reducing equivalent generated via the niacin pathway, directly supports antioxidant systems, including the glutathione cycle and thioredoxin system. Niacin thereby enhances AOC through NAD(P)H‐dependent antioxidant systems, modulation of oxidative stress pathways, and anti‐inflammatory effects [36]. It was demonstrated in rat that moderate niacin intake exhibits significant effects in both reducing liver transaminase levels and ameliorating hepatic steatosis [37]. In fish, hybrid sturgeon fed diets with ≥30 mg/kg niacin exhibited significantly increased total SOD activity and total AOC (T‐AOC) in muscle tissue [22]. Similarly, an appropriate amount of dietary niacin (120–160 mg/kg) significantly increased the activities of SOD and glutathione peroxidase in Pacific white shrimp (*Litopenaeus vannamei*) [38]. In grass carp, deficiency of niacin compromised cellular antioxidant defenses through NAD^+^‐dependent mechanisms: diminished NAD^+^ bioavailability attenuates SIRT3 and SIRT5 deacetylase activities, resulting in significant suppression of SOD activity, mitochondrial ultrastructural abnormalities, and impaired redox homeostasis in the skeletal muscle [39]. Building on these findings, our results suggested that niacin exerted its hepatoprotective effects in Nile tilapia partly through NADPH‐mediated reinforcement of the glutathione redox cycle. Because in the present study, Nile tilapia supplemented with 50–100 mg/kg niacin exhibited significantly lower serum alanine aminotransferase (ALT) activity—a marker of liver injury—compared to both deficient (0 mg/kg) and excessive (200 mg/kg) supplementation groups. Concurrently, hepatic SOD and CAT activities were significantly higher, along with upregulation of the glutathione S‐transferase (*gst*) gene in the moderately (100 mg/kg) supplemented group. These responses indicated that niacin sufficiency enhanced the enzymatic antioxidant system, thereby mitigating oxidative stress and preserving hepatocyte integrity. These results collectively demonstrated that niacin within the optimal range (50–100 mg/kg) effectively improved liver health, and enhanced AOC in Nile tilapia, whereas deviation from this optimal range—whether deficient or excessive—led to metabolic dysfunction and oxidative impairment.

## 5. Conclusions

The present study showed that dietary niacin deficiency led to growth inhibition in tilapia. However, excessive niacin intake (200 mg/kg) led to hepatic TG accumulation, which was associated with enhanced lipogenesis and suppression of lipid catabolism. Additionally, niacin deficiency induced hypoglycemia and reduced glucose metabolism activity in Nile tilapia. Consequently, both insufficient and excessive niacin intake compromised liver health and attenuated AOC in Nile tilapia. Our findings enable formulated feeds that boost tilapia robustness and profitability. The recommended niacin level (50–100 mg/kg) improves stress resilience and growth under intensive farming, directly enhancing sustainability and economic returns.

## Conflicts of Interest

The authors declare no conflicts of interests.

## Author Contributions

**Ruixin Li:** writing – original draft, supervision, project administration, methodology, funding acquisition, conceptualization. **Jingwen Liu:** writing – original draft, methodology, investigation, data curation. **Jiayong Liang:** methodology, investigation, data curation. **Lang Liang:** investigation. **Zexuan Kong:** investigation. **Tan Liu:** investigation. **Xiaojuan Liu:** writing – review & editing. **Fan Lin:** writing – review & editing. **Cuiying Chen:** writing – review & editing. **Zhenyu Du:** writing – review & editing, resources. **Shuqi Wang:** writing – review & editing, resources, funding acquisition. Jingwen Liu contributed equally as first author.

## Funding

This research was financially supported by Basic and Applied Basic Research Foundation of Guangdong Province (2024A1515110025), STU Scientific Research Initiation Grant (NTF23031), the National Natural Science Foundation of China (32373146), and Guangdong Agriculture Research System (2023KJ150).

## Supporting Information

Additional supporting information can be found online in the Supporting Information section.

## Supporting information


**Supporting Information** The completed ARRIVE Essential 10 checklist is provided as a supporting file.

## Data Availability

The datasets generated during the current study are available from the corresponding author on reasonable request.
